# Dental Teaching

**Published:** 1867-05

**Authors:** 


### Editorial.

### DENTAL TEACHING.

The work of properly instructing those who are to assume the duties and responsibilities of the Dentist, is one of no easy accomplishment. The fact that there is as yet no well defined and generally approved curriculum of study, is by no means one of the minor difficulties.

This is due no doubt to some extent to the fact that only a short time has elapsed since an effort was made to methodically and systematically teach the principles upon which the practice of our profession is based; perhaps, as much has been done as could have been expected in twenty-five years; this period embraces the beginning of the effort for systematic Dental teaching, and that effort from the very nature of circumstances could not be otherwise than limited, and partial in its results.

There had nothing in the form of a similar work gone before it, to break the path or dispel the darkness that covered the way.

With scarcely anything like a text-book, and no periodical literature, which should serve as free channels for the outflow and the exchange of thought, ideas and practice. With no Associations to encourage and cheer the ongoing in the weary way. Add to this the fact, that at first everything was experimental, no one could have in his mind, definitely what was best, or even what was needed, and this could not be determined, except by experiment wrought out through patient perseverence. And exceedingly fortunate was it for the profession, that in this time of need, it had such men as Hayden and Harris, and others we might name, some of whom are still with us, who labored with an energy, perseverence and efficiency, answerable to the requirements of the time, and for which they should ever be held in grateful remembrance by the profession. While we would not be hero worshipers, let us not manifest base ingratitude by hastily forgetting, or refusing to honor, the names of those who have been our greatest benefactors, some of whom only ceased to labor when they sank beneath the verge of the grave.

And though the difficulties mentioned above, have been to a large extent removed, there are others still operative. A want of the proper conception of what is necessary, to well prepare the Dentist for his legitimate work, has been, and still is, if not an actual obstacle—a dead weight, that much impedes true progress.

Many in the profession, and the vast majority of those not in the profession, imagine that mechanical tact and skill, and especially if one is much favored by nature—is all that is necessary in the way of endowments and attainments, to constitute a "splendid Dentist."

The misconception is based upon a want of knowledge and proper consideration. With such false and narrow views, of course very little encouragement will be given to the institutions and persons aiming to give and foster a liberal and properly extended professional education.

But further upon this subject in the next number.

